# Using Acoustic Signal and Image to Achieve Accurate Indoor Localization

**DOI:** 10.3390/s18082566

**Published:** 2018-08-06

**Authors:** Rui Xi, Daibo Liu, Mengshu Hou, Yujun Li, Jun Li

**Affiliations:** 1 School of Computer Science & Engineering, University of Electronic Science and Technology of China, Chengdu 611731, China; ruix.ryan@gmail.com (R.X.); dbliu.sky@gmail.com (D.L.); liyujun@uestc.edu.cn (Y.L.); 2 School of Computer Engineering, Chengdu Technological University, Chengdu 611731, China; suiyuanlj2006@gmail.com

**Keywords:** acoustic signals, images, indoor localization, smartphone, Internet of Things

## Abstract

Location information plays a key role in pervasive computing and application, especially indoor location-based service, even though a mass of systems have been proposed, an accurate and practical indoor localization system remains unsettled. To tackle this issue, in this paper, we present a new localization scheme, *SITE*, combining acoustic *S*ignals and *I*mages to achieve accurate and robust indoor loca*T*ion s*E*rvice. Relying on a pre-deployed platform of acoustic sources with different frequencies, using proactively generated Doppler effect signals, *SITE* could track relative directions between the phone and the sources. Given *m* (m≥5) relative directions, *SITE* can use the angle differences to compute a set of locations corresponding to different subsets of sources. Then, based on a key observation—while the simultaneously estimated locations using different sets of acoustic anchors are within a small circle, the results converge to a point near the true location—*SITE* proposes a decision scheme that confirms whether these locations satisfy the demand of localization accuracy and can be used to search the user’s location. If not, *SITE* utilizes VSFM(Visual Structure from Motion) technique to achieve a set of relative locations using some images captured by the phone’s camera. By exploiting the synergy between the set of relative locations and the set of initial locations computed by relative directions, an optimal transformation relationship is obtained and applied to refine the initial calculated results. The refined result will be regarded as the user’s location. In the evaluation, we implemented a prototype and deployed a real platform of acoustic sources in different scenarios. Experimental results show that *SITE* has excellent performance of localization accuracy, robustness and feasibility in practical application.

## 1. Introduction

During the past decade, as one of the key techniques of indoor location-based services (ILBS), accurate and inexpensive indoor localization problem has attracted a great deal of attention from academia and industry. Meanwhile, a mass of efforts and resources have also been devoted. Even thoguh many different indoor localization methods [1,2,3,4] have been proposed, this problem remains unsettled.

In this paper, we categorize the existing localization schemes primarily into two sets: fingerprinting-based and ranging-based. The former achieves indoor localization result by matching fingerprinting to a database; the fingerprint usually consisting of some existing indoor signals, such as WiFi [5,6], FM and TV [7], GSM [8], geo-magnetic [9], or sound signals [10,11]. However, site-survey, an essential component of building a fingerprinting database, is a time-consuming and labor-intensive task. Furthermore, due to the influence of environmental dynamics, the fingerprinting database should be updated frequently. For the ranging-based approaches, accurately estimating indoor location requires a pre-deployed platform of custom hardwares such as bluetooth beacons [12,13], magnetic resonators [14], ultrasound speakers [15], and custom RF transmitters [16]. However, to achieve high accuracy, a deployment of sophisticated and expensive anchors is required to calculate important information for location estimation, such as ToF [2,17], AoA [3] etc., which imposes extra costs, and is unsuitable for consumer device. Meanwhile, the inevitable instability of signals and synchronous error in indoor environments will also damage the robustness and availability of locations. To resolve these problems, complex algorithms are required, resulting in high computation costs and battery consumption, which are tremendous challenges for the memory and computation limited devices.

As is well-known, an ideal indoor localization system should satisfy the following four conditions: (1) the system should be deployed once and for all; (2) it can be constructed using off-the-shelf devices; (3) the cost is low and it is easy to deploy; and (4) it can consistently provide accurate and reliable location information. However, to achieve these goals is not-trivial. Overall, fingerprinting-based approaches cannot satisfy the first, third, and fourth conditions, while the ranging-based approaches cannot satisfy the second and third conditions.

For the widely used WiFi signals, due to the interference of indoor environments, pure WiFi-based localization can achieve reasonable accuracy (e.g., 3–4 m), but there always exist large errors (e.g., 6–8 m), which are unacceptable for many scenarios. Many additional RF signals, such as Bluetooth, ultrasound, etc., have been utilized to improve accuracy; however, the creation and updating of fingerprinting database is time-consuming and labor-intensive. Besides, while the layout of environment changes, it is still a critical issue to make the system stable and quickly resume. Although many ranging-based WiFi localization system have been proposed and achieved a high accuracy, additional specialized hardware is often required, which incurs much costs and is not suitable for large-scale scenario. The need of additional modulated device also violates the principles of a ubiquitous application. Contrarily, acoustic-based localization has less stringent requirements on timing accuracy, and can be widely deployed to the commercial off-the-shelf (COTS) smartphones, which are equipped with at least one speaker and one microphone. Moreover, it also provides a higher localization accuracy under a low-cost infrastructure. Hence, in this paper, we choose acoustic signals for indoor location determination. However, due to the existence of many interference factors, acoustic-based localization methods have a worse performance on robustness. For example, as mentioned in Section 3.2, there is a huge difference in accuracy when acoustic-based indoor localization method runs in different NLOS situations. According to the previous works, image-based localizations are impressively accurate at inferring relative distances and directions, and constructing a rigorous space relationship poses an opportunity to enhance the robustness and accuracy of acoustic-based localization methods.

In this paper, we propose *SITE*, a novel scheme that uses acoustic *S*ignal and phone *I*mages to achieve accurate and reliable indoor loca*T*ion syst*E*m. *SITE* uses fixed acoustic anchor to transmit acoustic signals that are inaudible to human but decodable by smartphone. Using proactively generated Doppler signals in rough horizontal plane, it can track the relative direction between the smartphone and the acoustic source. Hence, given a set of acoustic sources (more than 5), using the angle differences between relative directions, *SITE* could compute a set of locations, each corresponding to a subset of sources, whose size should be more than 2. Then, according to a key observation—while the simultaneously estimated locations using different sets of acoustic anchors are within a small circle, the results converge to a point near the true location—*SITE* proposes a decision scheme that confirms whether the estimated locations meet the accuracy requirement. According to this scheme, for any set of acoustic sources, if it and all of its subsets have a standard deviation below to a pre-defined threshold, then we can regard its corresponding localization result as the user’s location. Otherwise, through taking some images by the phone’s camera, *SITE* can utilize VSFM (Visual Structure from Motion) technique to achieve a set of relative locations. By exploiting the synergy between the set of relative locations and the set of initial locations computed by relative directions, an optimal transformation relationship is obtained and applied to refine the initial location. The refined result is regarded as the user’s location. By combining the Doppler effect, the new observation, and VSFM technique, *SITE* can not only achieve the angle-based localization system using low-cost mobile phone, but also guarantee the accuracy and simplify the deployment of anchor nodes.

We implemented a prototype and ran *SITE* in real mobile phoneswhich have microphone, a camera, and Yei Technology motion sensors. In the implementation, we utilized the VSFM [18] toolkit to obtain the camera relative locations. In the evaluation, we deployed two platforms with the same settings in a large building lobby and a large university library. From the experimental results and statistics, we found that *SITE* can achieve a median localization error of 0.42 m in Non-line-of-sight (NLOS) condition and 0.39 m in Line-of-sight (LOS) condition. In addition, they also indicate that *SITE* achieves a median improvement of localization accuracy of 54.67% and 43.83% compared to the state-of-the-art Swadloon [19], respectively. Besides, *SITE* has much more robust performance.

The contributions of this work are as follows:

- Through exploiting the characteristics of results computed by acoustic-based localization method, we propose a decision scheme to distinguish the deviated results from accurate localization. This mechanism not only increases the chance to tolerate signal instability of individual anchors, but also simplify the deployment of acoustic anchors.
- Based on the proposed decision scheme, we present *SITE*, a ready-to-use indoor localization system that can accurately infer the user’s location. To the best of our knowledge, no similar work has been done to exploit the features of acoustic signal and phone image yet.
- We implemented a prototype of *SITE* running on Android platform by utilizing the VSFM toolkit. Through comparative evaluation, we prove that *SITE* can achieve accurate and reliable location in many different conditions.

The rest of the paper is organized as follows. In Section 2, we first give a discussion about the related works. Next, in Section 3, we introduce some necessary preliminary knowledge, Meanwhile, we also explain and validate our observation. Subsequently, the detailed design of *SITE* is separately presented in Section 4. We describe the experimental settings and make a comparatively analysis of evaluation results in Section 5. Section 6 discusses some potential concerns and further works. Section 7 concludes the work of this paper.

## 2. Related Work

### 2.1. Indoor Direction Finding

To be a method for localization, Angle of Arrival (AoA) measurement has been utilized in many different localization systems. According to the way of measuring AoA, we classify these methods into two categories. One class needs special devices, such as directional antenna and antenna array, to implement AoA measurement in localization systems [1,3]. For example, using the directional antenna, the direction of AP, which is with the highest received strength, can be obtained only relying on rotating the antenna’s beam. For the antenna array [1,20], because each antenna receives the signal in an asynchronous mode, given the distance differences between antennas, using the time differences can compute AoA measurements. To measure AoA, another class requires smartphones rather than specialized hardware. For example, Zhang, et al. [21] used a phone to emulate a directional antenna, rotating it around the user’s body can pinpoint the direction of AP. In addition, according to the Doppler effect of acoustic signals caused by shaking the phone at different directions, by tracking the changing of the received frequency, Huang, et al. [19] estimated the phone’s direction relative to the acoustic source. Based on this work, they [22] made a further step to real-time localize and track the user.

### 2.2. Indoor Acoustic Localization

Comparing to using other signals for localization, acoustic-based localization is much easier to deploy in the commercial off-the-shelf (COTS) smartphones, and can achieve higher accuracy. Therefore, it has attracted a great deal of attention. For example, since a neighboring store in a shopping mall often offers a special service, it has a distinctive ambiance including sound, light, decor, etc. [10], the distribution of acoustic amplitude can be chosen as the fingerprint to discriminate neighboring stores. Besides, some methods utilize acoustic Doppler effect to infer the user’s location [22,23]. Based on the distribution of coordinates calculated by different subsets of acoustic sources, Xi et al. [23] proposed a validity judgment of location that generated by the acoustic-based localization method. Moreover, it put forward a refinement scheme combining with relative coordinates by VSFM technique to correct the invalid locations.

**Figure 1:**
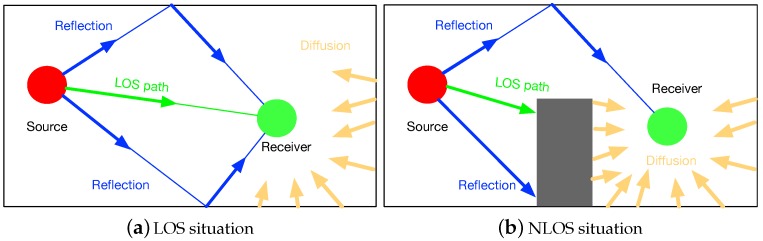
Line-of-sight (LOS) and non-line-of-sight (NLOS) situation [24].

Furthermore, facing the heavy NLOS problem (as illustrated in Figure 1) in a real indoor environment, Zhang et al. [24] proposed a way to identify and discard the NLOS measurements, resulting in improved localization performance. By analyzing acoustic propagations, it characterizes the difference of channel gain and channel delay between two propagation scenarios (NLOS and LOS) as the changes of acoustic channel, and leverages an SVM classifier to realize NLOS identification.

### 2.3. Image-Based Localization

Being an emerging technique, image-based localization was originally proposed in [25]. In this method, each building facade’s view will be associated with a 3D coordinate and stored in a database. The user can take an image and match it in the database to estimate pose. As the technology of Structure-from-Motion (SfM) advances, through reconstructing a 3D model can achieve accurate relative location. For example, Sattler et al. [26] presented a localization framework that directly matches the descriptors of 2D images to a 3D model. It simplifies the localization process and accelerates the efficiency. Many other methods have laos been presented. By detecting the edges of room in images, for each image, Kosecka et al. [27] generated an edge histogram and stored it in a database with its corresponding location information. To search the matched histogram of an image, the location is returned. Besides, Gao et al. [28] exploited Sextant to localize the user using its distance measurements relative to static reference objects, such as store logos. To eliminate image matching mistakes that will cause large localization errors, they also proposed a novel method to automatically identify reference objects from photos taken by a smartphone.

### 2.4. WiFi-Based Localization

As the most popular approach, fingerprinting-based WiFi indoor localization needs to build and update a fingerprinting database, which is full of the received signal strength indicator (RSSI) measurements at each known locations. Unfortunately, this is a time-consuming and labor-intensive task, and becomes a key bottleneck. To resolve these problems, many studies have been invested in infrastructure-free indoor localization. For example, to reduce human effort, Balzano et al. [29,30] proposed a framework to automatically and continuously update fingerprints. Based on an opportune deployment of a WSN, every sensor detects wireless data and sends to the server for updating RadioMap. This is robust to the network structure changes and environmental changes which will alter RSSs. The whole procedure does not require any human intervention. Along with the occasional location can be fixed by a GPS at the entrance or near a window, Chintalapudi et al. [31] exploited WiFi measurements to generate an initial probability distribution of the possible locations. Then, given distance constraints and distance-difference constraints obtained by acoustic measurements, using Bayesian can infer the most likely location. Once the GPS is available, the location is recalibrated. Furthermore, to eliminate distance error caused by the inaccurate propagation model, Kumar et al. [32] presented an efficient compartmental attenuation model to track node with multi-sensor data, and utilized a modified Prony estimator for high tracking accuracy. In addition, they developed a range-free method to estimate location, which significantly improves the convergence speed of localization.

Besides, much research has been invested in fusing WiFi and other RF signals. Kanaris et al. [33] introduced a hybrid method to improve accuracy by combining Bluetooth Low Energy (BLE) and WiFi. Based on the proximity of the BLE devices and a WiFi fingerprint dataset, They proposed i-KNN to extract an optimized subset of possible locations for localizing the user. Sergio et al. [34] utilized a WiFi map and an ultrasound map to infer the user’s location. They compared WiFi measurements with WiFi map to get an initial location, and uses a particle filter to propagate location with different weights acquired by ultrasound values. To remove errors caused by the blocking effect of the human body, Kessel et al. [35] utilized the user’s orientation from the compass to obtain a subset of fingerprints that contains those with a maximal deviation of $50^{\circ }$ from the orientation. Then, a weighted kNN is applied search the optimal location.

Table 1 presents a comparative analysis of many different localization systems from three aspects: accuracy, techniques and limitations. As discussed above, fingerprinting-based localization methods perform worst, only achieving meter-level accuracy. Using acoustic signals provides indoor localization with cm-level resolution, such as GuoGuo and Swadloon. Even though ranging-based WiFi localization methods can also perform a closer accuracy, the limitations make them unsuitable for large-scale deployment. For example, SpotFi uses some antennas to obtain AoA and ToF, but it requires the user’s device to continuously emit signals, which will can drain the device’s limited battery. Besides, it also requires time synchronization for estimating ToF. Meanwhile, because it cannot calculate location with limited number of signals, SpotFi is not suitable for real-time localization. ArrayTrack relies on comparatively larger number of antennas to calculate AoA at the WiFi AP, which is the fundamental limitation. In contrast, acoustic signals can provide higher accuracy, lower battery consumption, and easier large-scale deployment in indoor environment. However, e some limitations exist in acoustic-based localization systems. For example, GuoGuo requires customized acoustic beacons around the building. The shorter range of acoustic signals, and the limitation that cannot work in high sound pollution make GuoGuo unsuitable for a ubiquitous localization system. However, without any additional customized beacons and laborious operation, Swadloon provides a relative direction finding scheme that only relies on a pre-deployed beacons. By tracking the changing of the received frequency, it just requires the user to shake the phone for collecting Doppler signals. These characteristics give us an opportunity to localize the user by acoustic signals. However, affected by the sound pollution in indoor environment, it has unstable performance when calculating location. To tackle this issue, based on Swadloon, we propose a ubiquitous and scalable indoor localization system, SITE, which is detailed in Section 4.

This paper is an extension work based on the previous paper [23] accepted by the conference ICPADS 2016. The main differences are listed as follows,

1. Based on [23], to enhance the stability and accuracy of acoustic-based localization method, we make a deeper and more comprehensive analysis of characteristics of the estimated results. A detailed explanation is described in Section 3.2.
2. According to our observation in Section 3.2, to build a fault-tolerant, highly reliable localization systems, we revise the module *Decision Scheme*. Instead of directly comparing deviation to a threshold δ, we propose a algorithm for searching a ConvergenceSet, that each subset satisfies our observation; a detailed description is given in Section 4.3.
3. In the evaluation, we compared the localization performance with [23] and Swadloon; the experimental results are shown in Section 5.2. Meanwhile, we also performed a complementary experiment on overhead, as described in Section 5.2.5.

## 3. Preliminary and Observation

In this section, firstly, we give an introduction to calculate the phone’s relative direction according to the Doppler effect. Then, we present a preliminary experiment, and, based on the results, we give a key observation that the diversity of estimated results indicates its difference between the real physical location. Based on it, a novel localization method combining acoustic signal with image processing is presented in the next section.

### 3.1. Proactive Acoustic Direction-Finding

Suppose that an acoustic source is emitting sinusoidal signal at frequency $f_{s}$. $v_{r}$ is a receiver’s moving velocity, which is positive when the receiver is moving towards the source, otherwise it is negative. $v_{s}$ and $v_{a}$ denote the moving velocity of acoustic source and the spreading speed of sound in air, respectively. Based on the Doppler effect, the received frequency $f_{r}$ is:

(1) $$f_{r}=\frac{v_{a}+v_{r}}{v_{a}+v_{s}}\cdot f_{s}$$

If the source keeps still or $v_{s}\ll v_{r}$, we can obtain the frequency shift $f_{shift}\approx \frac{f_{s}}{v_{a}}v_{r}$. Meanwhile, assuming that the received signal is

(2) $$r\left(t\right)=A\left(t\right)\cos(2\pi f_{s}t+\theta \left(t\right))+\delta \left(t\right)$$

where, A(t), θ(t), and δ(t), respectively, denote amplitude, phase and noise. Note that the amplitude A(t) changes continuously, and the phase θ(t) is affected by Doppler effects. Hence, the observed frequency shift $f_{shift}$ at time *t* can represented as

(3) $$f_{shift}\left(t\right)=\frac{1}{2\pi }\frac{d(2\pi f_{s}t+\theta \left(t\right))}{dt}-f_{s}=\frac{1}{2\pi }\frac{d\theta (t)}{dt}$$

Then, according to equations mentioned above, using observing the changing of received frequency, we can get the relative velocity v(t) between the phone and acoustic source and and the phone’s relative displacement s(t)

(4) $$\begin{array}{c} \begin{array}{ccc} v(t) & = & \frac{v_{a}}{2\pi f_{s}}\frac{d\theta (t)}{dt}\\ s(t) & = & \frac{v_{a}}{2\pi f_{s}}\theta \left(t\right)-\frac{v_{a}}{2\pi f_{s}}\theta \left(0\right) \end{array} \end{array}$$

Let L(t) represent the distance between the phone and acoustic source at time *t*, so s(t) = L(0) − L(t). Therefore, to get precise velocity and displacement, we have to track the phase θ(t) within a tiny error.

Because we are only interested in the 2D direction α rather than the 3D direction $\left(\lambda_{x},\lambda_{y},\lambda_{z}\right)$, $\lambda_{z}$ is not needed during the direction finding phase; therefore, suppose that the phone moves in a horizontal plane that $\lambda_{z}$ is zero, for a given velocity vector of the phone $\vec{u}=\left(v_{x},v_{y},v_{z}\right)$ and f[k],

(5) $$\lambda_{x}v_{x}\left[k\right]+\lambda_{y}v_{y}\left[k\right]=\frac{v_{a}}{f_{a}}\cdot f\left[k\right]$$

where $\lambda_{z}v_{z}\left[k\right]\approx 0$. According to Equation (5), we could eliminate the error of $v_{z}$ and obtain $\lambda_{x}$ and $\lambda_{y}$ using linear regression (LR) algorithm. Consequently, the 2D direction α is calculated by

$$\alpha =\left\{\begin{array}{cc} \arcsin\frac{\lambda_{y}}{\sqrt{\lambda_{x}^{2}+\lambda_{y}^{2}}} & \lambda_{x}\geq 0\\ \pi +\arcsin\frac{\lambda_{y}}{\sqrt{\lambda_{x}^{2}+\lambda_{y}^{2}}} & \lambda_{x}<0 \end{array}\right.$$

Hence, by proactively tracking Doppler signals, we can compute the real-time relative direction between an individual acoustic source and mobile phone. In Section 4, we present *SITE* that utilizes a set of relative directions between the phone and the pre-deployed acoustic sources to localize the user’s location.

### 3.2. Observation

In the indoor environment, many interference factors can influence localization result. These factors include moving people, multi-path interference, background sounds, etc. If calculated result is far away from the real physical location, it is regarded as false. In a practical localization system, it is vital to avoid using false location. However, without the real physical location, judging whether the estimated location is near the real physical location is still an open issue.

**Figure 2:**
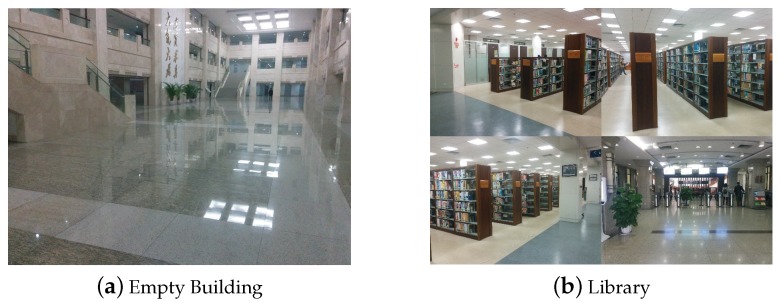
Experiment Environments.

**Figure 3:**
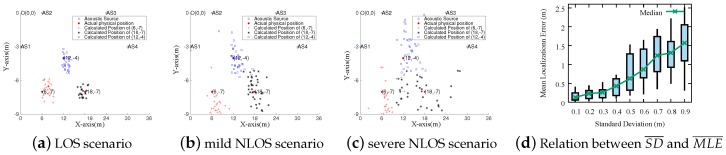
Experimental results.

To figure out the relationship between estimated locations and real physical location, we used the prototype of acoustic-based localization method proposed in [19] to conduct extensive evaluations at three indoor conditions: LOS, mild NLOS and severe NLOS. LOS represents a scenario that no acoustic source is blocked. When fewer than three sources are blocked, we deem this scenario to be mild NLOS. Accordingly, we define severe NLOS situation that has more than three acoustic sources are blocked. Moreover, as shown in Figure 2, our evaluations were conducted in two indoor circumstances: building lobby and library. We repeat the localization process at three fixed points under different levels of noise interference. The setting of acoustic anchors and the located points are illustrated in Figure 3. We pre-deployed four acoustic sources at (0,−3), (6,0), (24,0), and (30,−3), which are marked as diamonds in the figure. The coordinates of located locations at (6,−7), (18,−7) and (12,−4) are marked as red solid circles.

As Figure 3 illustrates, we can intuitively observe that there is a tremendous difference in accuracy between different indoor scenarios. Furthermore, these results reveal an interesting phenomenon: With the good condition of an indoor environment (see Figure 3a), the estimated locations are densely scattered over a relatively small area near the real physical location. On the contrary, with bad conditions (see Figure 3b,c), the estimated locations are scattered over a larger area and some estimated locations may be far from real physical location. Based on this observation, we assume that, if the simultaneously computed locations for the same physical location by using different acoustic sources change little, the localization result is very close to the real physical location. Otherwise, it probably deviates from the real physics location. We prove the assumption by conducting extensive experiments as presented in the following.

Assuming *M* coordinates {($x_{1},y_{1}$), ($x_{2},y_{2}$), ⋯, ($x_{M},y_{M}$)} correspond to the same point (x,y); then, we can compute their standard deviation ($\bar{SD}$) according to Equation (6).

(6) $$\bar{SD}=\frac{1}{M}\sum_{i=1}^{M}\sqrt{{\left(x_{i}-\bar{X}\right)}^{2}+{\left(y_{i}-\bar{Y}\right)}^{2}}$$

where we define $\left(\bar{X},\bar{Y}\right)$ as the mean coordinate of all the computed candidate coordinates. In addition, we further compute the mean localization error ($\bar{MLE}$) as Equation (7).

(7) $$\bar{MLE}=\frac{1}{M}\sum_{i=1}^{M}\sqrt{{\left(x_{i}-x\right)}^{2}+{\left(y_{i}-y\right)}^{2}}$$

According to our assumption, $\bar{MLE}$ will be positively correlated with $\bar{SD}$. As the standard deviation increases, the average localization error gets larger correspondingly. For each experiment mentioned-above, we repeat 10 times at the different time of a day. For each experiment, we plot the pair of $\bar{SD}$ and $\bar{MLE}$ in Figure 3d. As demonstrated, $\bar{MLE}$ is positively related to $\bar{SD}$: $\bar{MLE}$ increases with rising $\bar{SD}$. For each $\bar{SD}$, we compute the distribution of $\bar{MLE}$ and plot the maximum value, 3rd quartile, median value, 1st quartile and the minimum value of $\bar{MLE}$s. As shown by the figure, the larger the $\bar{SD}$ is, the larger range the $\bar{MLE}$s are distributed in. Note that the diversity of localization results directly affect both $\bar{SD}$ and $\bar{MLE}$. For a given set of locating results, by restricting the value of $\bar{SD}$, e.g., smaller than 0.3, if there exists a subset of results satisfying the restriction, the corresponding $\bar{MLE}$ will probably be very small, which indicates the subset of locations closely match the physical location. This phenomenon is confirmed in Figure 3d and it is consistent with our expectation.

**Figure 4:**
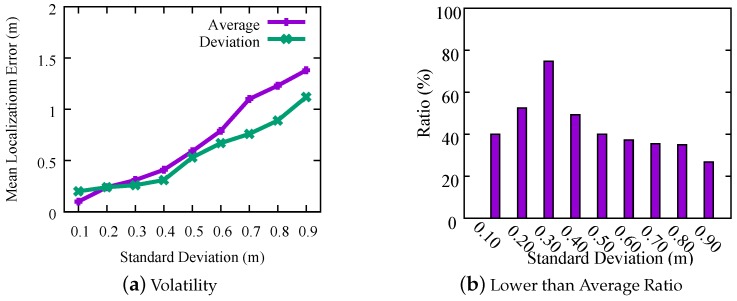
Further analysis on performance of $\bar{MLE}$.

Moreover, for each $\bar{SD}$, we also compute the averaged $\bar{MLE}$ and the volatility of localization results by $average=\frac{1}{N}\sum_{i=1}^{N}{\bar{MLE}}_{i}$ and $deviation=\frac{1}{N}\sum_{i=1}^{N}\sqrt{{\left({\bar{MLE}}_{i}-average\right)}^{2}}$ of $\bar{MLE}$. The averaged $\bar{MLE}$ denotes the overall location accurate when all estimated results satisfy the given restriction of $\bar{SD}$, and the deviation denotes the volatility of all localization results. As shown by Figure 4a, the averaged $\bar{MLE}$ increases with the rising of $\bar{SD}$, and the volatility of $\bar{MLE}$ first steadily increases when $\bar{SD}$ increases from 0.1 m to 0.3 m, and then significantly increases from 0.2 m to 1 m when $\bar{SD}$ increases from 0.3 m to 0.9 m. In addition, for a given $\bar{SD}$, we further compute the probability that the actual $\bar{MLE}$ of each calculated result is less than the given $\bar{SD}$, and plot the distribution in Figure 4b. As shown by it, when $\bar{SD}$ is 0.3m, the actual $\bar{MLE}$s of about 80% results are less than 0.3 m. The probability of $\bar{SD}=$ 0.3 m is significantly higher than other cases. Based on these results, in the *Decision Scheme* (see Section 4.3), we set the threshold δ to 0.3.

With all observations mentioned above, we design a smartphone-based indoor localization system, *SITE*, that can judge the usability of the estimated location and refine it when it is unusable. The next section introduces the details of our proposed design.

## 4. Design of SITE

In this section, we give the detailed design of *SITE*. We first introduce the overview of *SITE* in Section 4.1. On the basis of acoustic Doppler effect, we use acoustic anchors to compute the physical location of a mobile phone in Section 4.2. According to a set of computation results, SITE determines whether the estimated location can represent the real physical location in Section 4.3. If not, *SITE* uses VSFM technique to refine the estimated result in Section 4.4.

### 4.1. Overview of SITE

**Figure 5:**
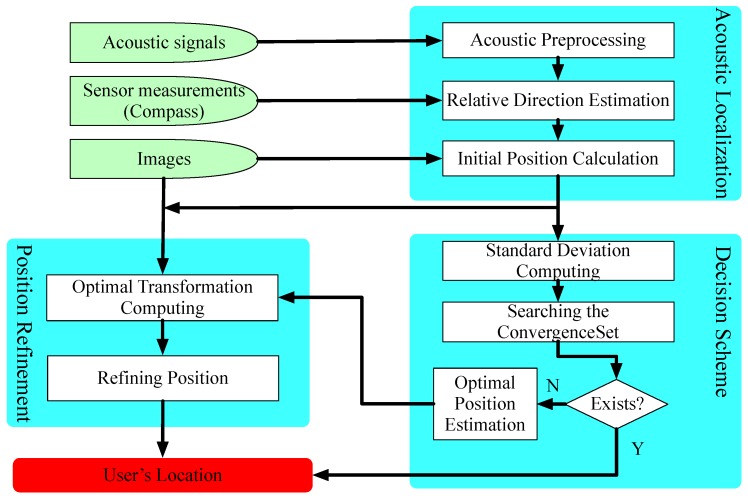
The architecture of *SITE* consists of three modules: *Acoustic Localization*, *Decision Scheme*, and *Position Refinement*.

As shown in Figure 5, *SITE* contains three main components:

- *Acoustic Localization* is a module that can localize user (mobile phone) using relative directions between the phone and acoustic sources according to acoustic Doppler effect. This model consists of three sub-modules: *Acoustic Preprocessing*, *Relative Direction Estimation* and *Initial Position Calculation*. Given acoustic signals, *Acoustic Preprocessing* first eliminates the interference and adjusts the amplitude. Then, *Relative Direction Estimation* estimates relative direction between device and each acoustic anchor based on the theory of Doppler effect. With directions relative to a set of anchors, *Initial Position Calculation* computes a set of initial locations, each corresponding to a different set of relative directions, to find the optimal. In Section 4.2, we give a detailed introduction of this module.
- *Decision Scheme* is a module that assesses the accuracy of localization result. It consists of sub-module *Judgement Condition* and *Finding the Optimal Coordinate*. In sub-module *Judgement Condition*, *SITE* judges the state (*CONVERGED* or *DIVERGED*, see Section 4.3.1) of a set of initial locations calculated by *Acoustic Localization* according to our observation introduced in Section 3.2. Then, if the state is *CONVERGED*, sub-module *Finding the Optimal Coordinate* is activated to search for an optimal coordinate for the device. We give detailed design of this module in Section 4.3.
- *Position Refinement* is a module that can refine the localization result with images by VSFM technique. We introduce it in Section 4.4.

With a pre-deployed platform of acoustic sources that emit the sinusoid signals at a different specific frequency, module *Acoustic Localization* first eliminates interference signals and adjusts amplitudes of received signals through acoustic preprocessing technology. Then, it estimates the phone’s direction relative to an individual acoustic source via the above-mentioned method. The initial location is a set of relative directions and a set of acoustic sources with known coordinate. Note that *Acoustic Localization* could simultaneously obtain a set of locations, each corresponding to a different set of acoustic sources. According to our observation and proposed principle mentioned in Section 3.2, the sub-module *Decision Scheme* computes the $\bar{SD}$ of these calculated locations. Subsequently, it judges whether the phone has been accurately localized by comparing the Standard Deviation with a pre-measured threshold in Section 3.2. If so, the user’s location can be achieved by *Initial Position Calculation*. Otherwise, the module *Location Refinement* is activated to refine the computed uncertain location through VSFM technique. In the following sections, we give a detailed introduction of these modules.

### 4.2. Acoustic Localization

Here, we introduce how to compute the initial location by finding the acoustic source’s direction relative to smartphone. As illustrated in Figure 5, it consists of three procedures: *Acoustic Preprocessing*, *Relative Direction Estimation* and *Initial Position Calculation*. In our implementation, we make a brief reference to the work in [19] to estimate the relative direction. Next, we simply introduce its procedures in *Acoustic Preprocessing* and *Relative Direction Estimation*. Besides, we describe the principle and procedure of calculating the initial location with these relative directions.

#### 4.2.1. Acoustic Preprocessing

In acoustic-based localization system, interference includes other acoustic waves that generated by other mobile phones or other acoustic sources. We denote external interference as δ(t) in Equation (2). To eliminate these interferences, we first pass the received signals r(t) through a Band Pass Filter (BPF) that only the signal at a specific frequency will pass. Consequently, signals from other sources and low frequency noises are eliminated. Hence, the acoustic signals can be represented using Equation (8):

(8) $$r^{\prime }\left(t\right)=A\left(t\right)\cos\left(2\pi f_{s}t+\theta \left(t\right)\right)$$

In addition, to avoid resulting in distortion of the different frequency component, we choose the equiripple FIR filter as our ideal BPF in the prototype of SITE.

Subsequently, we adjust the filtered acoustic signals $r^{\prime }\left(t\right)$ by Automatic Gain Control (AGC) that results in modify the the amplitude A(t) to (almost) a constant. Eventually, we get the acoustic signals $r^{\prime }\left(t\right)=\cos\left(2\pi f_{s}t+\theta \left(t\right)\right)$. Next, we describe how to precisely track the phase θ(t) by using PLL for estimating direction.

#### 4.2.2. Relative Direction Estimation

As mentioned in Section 3.1, we can estimate the relative direction between a device and an acoustic anchor by using LR algorithm to solve Equation (5). To do that, we should get the precise velocity and displacement in advance. Hence, we first utilize Phase Locked Loops (PLL) to track the changing of phase θ(t) while the device is moving. Then, we can get the precise displacement s(t) and velocity v(t) as shown in Equation (4). On that basis, we compute a 2D relative direction vector using a linear regression, and eventually compute the relative direction α in WCS (World’s Coordinate System), which can be acquired by compass.

**Figure 6:**
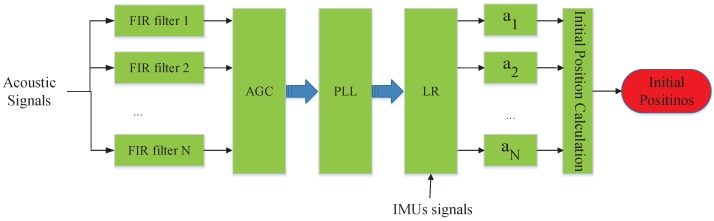
Procedure of *Acoustic Localization*. Acoustic signals firstly pass through FIR filter and AGC to eliminate interference. Then, PLL tracks the changes of the phase of received acoustic signals. The 2D relative directions ($\alpha_{1},\alpha_{2},\cdots ,\alpha_{N}$) are calculated via LR algorithm. Finally, *Initial Position Calculation* computes coordinate using relative directions.

Although we can estimate the relative direction between a device and an acoustic anchor, SITE needs to further calculate a set of relative directions using at least three acoustic sources to localize user. In a localization system, several acoustic anchors are pre-deployed. Hence, SITE needs to compute the relative directions between multiple nearby acoustic sources to the device simultaneously. As shown in Figure 6, the received acoustic signals parallel walk through many FIR filters (*FIR filter 1*, *FIR filter 2*, ⋯, and *FIR filter N*), each with different frequency bandwidth thresholds. The threshold value is set according to the frequency of pre-defined acoustic sources. Then, through sequentially processing by AGC, PLL and LR, the filtered signals by different FIR filter will generate a set of relative directions ($\alpha_{1},\alpha_{2},\cdots ,\alpha_{N}$). Eventually, with these relative directions, *SITE* can compute location using the difference of relative directions, we will give an introduction in the following section.

#### 4.2.3. Initial Position Calculation

**Figure 7:**
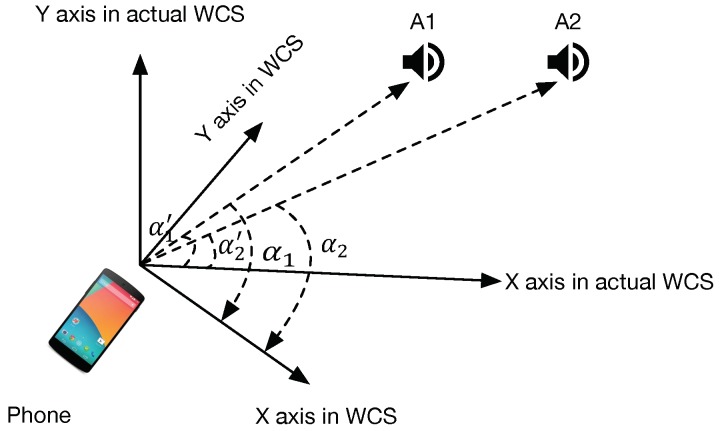
Illustration of the calculated WCS and the actual WCS. The calculated WCS is transformed by the User’s phone Coordinate System using the compass. The $\alpha_{1}$ and $\alpha_{2}$ represent the calculated relative directions in the WCS, and $\alpha_{1}^{\prime }$ and $\alpha_{2}^{\prime }$ are their corresponding actual relative directions in the actual WCS, respectively.

As mentioned above, the phone calculates the direction of each anchor node in WCS for calculating the location. However, the WCS is acquired by the compass, due to the error of compass; Figure 7 shows that the X axis in WCS may not point to the X axis in the actual WCS. Thus, the calculated relative direction $\alpha_{1}$ and $\alpha_{2}$ may not be the actual direction relative $\alpha_{1}^{\prime }$ and $\alpha_{2}^{\prime }$. In the figure, we can see that the difference $|\alpha_{1}-\alpha_{2}|$, also named as the opening angle, is fixed that equals to $|\alpha_{1}^{\prime }-\alpha_{2}^{\prime }|$. Its accuracy is not be affected by the interference from the compass. Hence, to remove the cumulative errors of the compass, *SITE* utilize the opening angle to estimate initial coordinates/locations.

**Figure 8:**
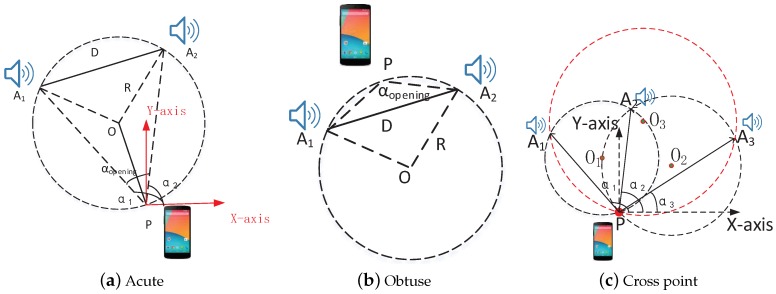
Location estimation: (**a**) *α_opening_* is acute; (**b**)*α_opening_* is obtuse; and (**c**) chosen location lies on the crossing of three circumcircles *O*_1_, *O*_2_, and *O*_3_ to be the phone’s location. *α*_1_, *α*_2_ and *α*_3_ represent the AoA estimations, which are relative to the phone.

In Figure 8a, there are two acoustic sources, $A_{1}$ $\left(x_{1},y_{1}\right)$ and $A_{2}$ $\left(x_{2},y_{2}\right)$, and their corresponding directions relative to mobile phone *P* (with unknown coordinate (x,y)) are $\alpha_{1}$ and $\alpha_{2}$. By computing the distance $D=\left|\right|A_{1}-A_{2}\left|\right|$ and the opening angle $\alpha_{opening}=\left|\alpha_{1}-\alpha_{2}\right|$, we can infer that *P* locates on a fixed circle, whose radius (*R*) can be calculated with the distance *D* and the opening angle $\alpha_{opening}$. Then, as $A_{1}$ and $A_{2}$ are known, we get two possible results for circumcenter *O*. If $\alpha_{opening}$ is an acute angle, the circumcenter *O* and *P* are on the same side of $A_{1}A_{2}$ as shown in Figure 8a. Otherwise, they are on the opposite side, as shown in Figure 8b.

However, as Figure 8c illustrates, while there are three acoustic sources ($A_{1}$, $A_{2}$, and $A_{3}$) and the corresponding relative directions are $\alpha_{1}$, $\alpha_{2}$, and $\alpha_{3}$, we get two cases of phone’s location using above method. One is that the calculated location lies on the crossing of three circumcircles ($O_{1}$, $O_{2}$ and $O_{3}$) as shown in Figure 8c. Thus, we refer to the crossing point as an initial location. Another is with many alternative points once that three circumcircles do not locate at one point. Therefore, we have to choose the optimal as an initial location. In addition, if the number of acoustic sources is more than 3, we can also compute the coordinate in a similar way.

As described above, if there are *N* pre-deployed acoustic sources, there will be at most $C_{N}^{2}$ circumcircles and $C_{N}^{3}$ possible coordinates. Then, we have to find the optimal coordinate from these alternative coordinates. However, the optimal coordinate should have a minimum distance to all the circumcircles, each corresponding to two selected acoustic sources and the mobile phone, as shown in Figure 8c. To search the optimal, we compute a accumulative distance D (D = $\sum_{i=1}^{C_{N}^{2}}d_{i}$), where $d_{i}$ represents the distance to an individual circumcircles’s arc. Then, the distance relative to a calculated location (with coordinate $\left(x^{\prime },y^{\prime }\right)$) can be calculated as follows,

(9) $$\begin{array}{c} d_{i}=\left|\sqrt{{\left(x^{\prime }-x_{centre_{i}}\right)}^{2}+{\left(y^{\prime }-y_{centre_{i}}\right)}^{2}}-R_{centre_{i}}\right| \end{array}$$

Here, ($x_{centre_{i}},y_{centre_{i}}$) and $R_{centre_{i}}$ represent the centre coordinate and radius of a given circumcircle, respectively. Eventually, the coordinate corresponding to minimum D is selected as the initial location.

By analyzing the calculation method, if taking all acoustic sources for calculating initial location, it is still evident that the computation workload is a very big burden for smartphone’s limited battery capacity, even though its computational capability has been greatly improved. Moreover, if all signals are used to compute location, it will bring in much more error that is likely to obtain a *NULL* by the module *Decision Scheme*, which is explained in Section 4.3. Then, the module *Location Refinement* is activated, incurring much additional computation costs. Therefore, a measurement should be taken to avoid incurring a huge computation burden as well as possible. Assuming *SITE* has *N* anchor nodes, we select M(3<M<N) anchors with the strongest signal. Then, there is $\sum_{3}^{M}C_{M}^{i}$ initial locations for the user. On a condition that the captured anchor nodes are less than *M* caused by signal attenuation, e.g., interference signals, *SITE* will adopt all of them to calculate initial location. Note that, if the number is fewer than 3, *SITE* fails to location the phone. Hence, for balancing the computational workload and localization accuracy, we set *M* as 6 in our implementation of *SITE*.

According to the observation mentioned in Section 3.2, with a set of initial coordinates, *SITE* can make sure whether the localization results are close to the real physical location within a threshold. If so, we can obtain a coordinate to regard as the user’s location. Next, we describe how to achieve it. Otherwise, *SITE* refines calculated coordinates by VSFM technique. We describe it in Section 4.4.

### 4.3. Decision Scheme

Due to many interference factors in an indoor environment, using Acoustic Doppler Effect to calculate coordinates mentioned in Section 3.2 could lead to low stability and availability. To tackle this, we present a novel scheme to judge whether a coordinate estimated by a set of acoustic sources satisfies the observation result presented in Section 3.2. Once it fails, module *Location Refinement* is triggered to refine the estimated result as the user’s location. For reducing the interference from other factors, we exploit a searching algorithm to compute the initial location. Next, we introduce the judgement scheme (Section 4.3.1) and the searching algorithm (Section 4.3.2) in detail.

#### 4.3.1. Judgement Scheme

Before describing the scheme, we firstly give the definition of DistributionState(DS). DS represents the state of a set of coordinates corresponding to a specific true physical coordinate, and it could be assigned to two states: *CONVERGED STATE* and *DIVERGED STATE*. *DIVERGED STATE* means that a set of coordinates deviates from its true location and cannot be directly used. Contrarily, we mark this set as *CONVERGED STATE* while we deem it to be convergence to its true location. According to our observation described in Section 3.2, given a set of coordinates (s) computed at same spot, we can compute its DS according to the following equation,

(10) $$DS_{s}=\left\{\begin{array}{cc} CONVERGED\;STATE & {\bar{SD}}_{s}<\delta \\ DIVERGED\;STATE & {\bar{SD}}_{s}\geq \delta \end{array}\right.$$

Here, ${\bar{SD}}_{s}$ represents the standard deviation, and it can be computed as Equation (6). δ is a fixed threshold and is referred to as *Decision Factor*. In the implementation, we choose δ to be 0.3 m.

In Section 3.2, through experiments, we observe that, when a set s has a $\bar{SD}$ less than 0.3 m, the localization error of more than 80% localization results is less than 0.2 m. If both of its subsets also have a $\bar{SD}$ less than 0.3 m, we consider that it could achieve the most accurate localization result. For simplicity, we define a notation ConvergenceSet to represent this kind of set. Based on this, we propose a novel scheme to decide whether there is an estimated coordinate that is accurate enough to represent the localization result. In other words, we need to search for the largest ConvergenceSet from the received acoustic sources in module Section 4.2.

Here, we present a bottom-up searching algorithm to find out the ConvergenceSet. Given a set of source anchors, $S^{M}$ (M ≥ 4, represents its size), splitting it to many subsets with different size, these subsets are denoted as $\left\{S_{i}^{m},m=4,5,\cdots ,M;i=1,2,\cdots ,C_{M}^{m}\right\}$, and we refer to it as $SS^{M}$. $S_{i}^{m}$ represents the *i*th subset at the size of *m*. As mentioned above, a set $S^{M}$ labeled as ConvergenceSet must satisfy three requirements: (1) M is larger than 4; (2) $DS_{S}^{M}$ is less than δ; and (3) each element of $SS^{M}$ is marked as *CONVERGED STATE*, or all the subsets $S^{M-1}$ are ConvergenceSet. The detailed searching procedure is given in Algorithm 1.

Through searching procedure, *SITE* computes a collection of ConvergenceSet. However, there are three results on the number of ConvergenceSet in CL: (a) more than 1; (b) only one; and (c) NULL. According to our observation, when there is only one ConvergenceSet, the estimated coordinate by this set can be regarded as the user’s physical location. For the other two conditions, we take the following measures to compute user’s physical location.

- For the case that there are more than one ConvergenceSet, according to the searching algorithm, we know that these ConvergenceSets have the same size. Furthermore, as explained in Section 3.2, when there are the same amount of anchors for localization, $\bar{MLE}$ and $\bar{SD}$ will perform a positive correlation to a certain extent. Therefore, we deem that a ConvergenceSet with least $\bar{SD}$ can achieve the most accurate localization result. Hence, *SITE* uses it to compute the user’s location. In addition, if there are more than one sets with the least $\bar{SD}$, which also means that these sets have same $\bar{MLE}$, the center point of their corresponding estimated coordinates is deemed to be the user’s location.
- For the other case, while an empty CL is returned, it reveals that the results estimated by module *Acoustic Localization* has been seriously affected by many indoor interference factors; thus, these results cannot be directly used. Moreover, no coordinate can be obtained according to the method mentioned above. Therefore, it is necessary to take a further step to estimate the user’s location.

**Algorithm 1:** Procedure of searching largest ConvergenceSet.**Input**: $S^{M}$, a collection of acoustic sources, *M* is the size; **Output**: CL, a collection of the biggest ConvergenceSet; 
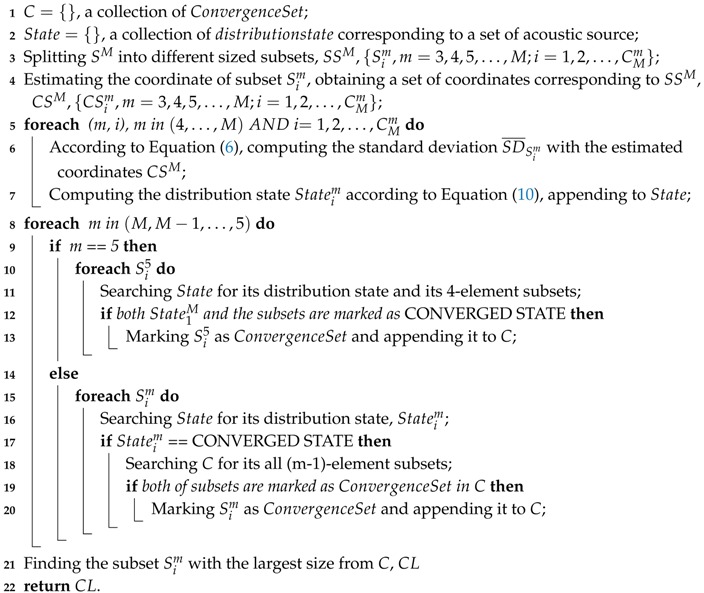


#### 4.3.2. Finding the Optimal Coordinate

On the consideration that no effective method could discriminate and remove interference sources, and using more acoustic sources to compute coordinate has a higher probability to bring in more interference, we employ some four-element sets of acoustic anchors to search the optimal coordinate, and these sets should be *CONVERGED STATE*. As above mentioned, in our implementation, we define the threshold δ to be 0.3 m for assessing a set’s distribution state, but, it will often result in a condition that no four-element set is available for computing initial coordinate. To prevent this condition, we bring in another threshold $\delta_{1}$ to reassess the distribution state of the four-element set. For balancing accuracy and availability, we choose $\delta_{1}$ to be 0.45 m, which is respect to a mean localization error of 0.5 m as Figure 4a shows.

Given *M* foursized sets marked as *CONVERGED STATE*, we generate *M* coordinates, $\left\{\left(x_{1},y_{1}\right),\ldots ,\left(x_{i},y_{i}\right),\ldots \right\}$, i=1,2,…,M. Coordinate $\left(x_{i},y_{i}\right)$ represents the estimated coordinate of *i*th set, therefore, finding an optimal initial coordinate will be transformed into a minimum optimization problem, and it should have minimum cumulative distance relative to all these coordinates. Based on the observations in Section 3.2, we assume that any set with different value of $\bar{SD}$, its corresponding estimated coordinate should have a different weight on the cumulative distance. Then, this minimum optimization problem can be expressed as Equation (11), and it could be resolved by searching an unknown coordinate (*x*, *y*) that minimizes the error in fit.

(11) $$\min_{x,y}\sum_{i=1}^{M}\epsilon_{i}\sqrt{{\left(x-x_{i}\right)}^{2}+{\left(y-y_{i}\right)}^{2}}$$

where $\epsilon_{i}$ is the weight of the estimated coordinate computed by *i*th set of acoustic sources. In this paper, we deem that the lower $\bar{SD}$ of a set is, the higher weight its corresponding coordinate has. For simplicity, we compute the weight $\epsilon_{i}$ using (12),

(12) $$\epsilon_{i}=\frac{1}{1+{\bar{SD}}_{i}}$$

${\bar{SD}}_{i}$ corresponds to the standard deviation of *i*th set. Therefore, combining Equations (11) and (12), the minimum optimization problem of finding an optimal coordinate can be represented as Equation (13),

(13) $$\min_{x,y}\sum_{i=1}^{M}\frac{1}{1+{\bar{SD}}_{i}}\sqrt{{\left(x-x_{i}\right)}^{2}+{\left(y-y_{i}\right)}^{2}}$$

In our implementation, we realize the gradient descent algorithm to solve this optimization problem. With this initial coordinate, *SITE* adopts VSFM technique to refine it to be the user’s physical location. We make a detailed introduction of refinement in the following section.

### 4.4. Position Refinement

Only relying on overlapping images, today’s vision techniques not only can reconstruct 3D point cloud, but also are impressively accurate at inferring relative distances and orientations. Based on this, we employ VSFM to technique to acquire relative coordinates for refining the initial coordinate that is estimated in module *Decision Scheme*. Then, the refined coordinate will be regarded as user’s physical location.

To get a precise three-dimensionl reconstruction for deriving the accurate camera’s relative locations, the user will be asked to take some photos. Following, after moving a few steps, s/he should repeat procedures of *Acoustic Localization* and *Decision Scheme*. It is noteworthy, that once *SITE* successfully gets ConvergenceSet in *Decision Scheme*, the module *Position Refinement* is not necessarily conducted. However, if *SITE* fails to find ConvergenceSet three times in succession, there are many images captured at *K* spots. With them, for each spot, *SITE* generates a relative coordinate using VSFM technique.

Accordingly, a user will have a set of pairs of two coordinates: (1) initial coordinate, namely optimal coordinate and detailed estimation is given in Section 4.3.2; and (2) relative coordinate, generated by VSFM technique with images captured by camera. Each pair of coordinates satisfies the transformation relationship (R,T) as Equation (14) shows,

(14) $$X_{g}=RX_{r}+T$$

where $X_{g}$ and $X_{r}$ represent a initial coordinate and a relative coordinate corresponding to a same spot, respectively. What the best situation is that there should be only one transformation exist. Unfortunately, for these pairs, the transformation relationships are different. Therefore, a suitable transformation between the initial coordinate and the relative coordinate should be found.

Assuming that there are *K* initial locations ${\vec{x}}_{g_{1}},\cdots ,{\vec{x}}_{g_{K}}$ and *K* relative locations ${\vec{x}}_{r_{1}},\cdots ,{\vec{x}}_{r_{K}}$, an optimal transformation relationship between these pairs of coordinates should minimize the result, as shown in Equation (15).

(15) $$\min_{r,t}\sum_{i=1}^{K}\left|\right|{\vec{x}}_{g_{i}}-\left(R{\vec{x}}_{p_{i}}+T\right){\left|\right|}^{2}$$

where the unknown transformation (R,T) is the key of the optimization problem. In general, the more pairs of coordinates we get, the more accurate a transformation relationship we can achieve. After obtaining the optimal transformation relationship, we can get the refined location as Equation (16)

(16) $${\vec{x}}_{global}=R{\vec{x}}_{relative}+T$$

where vectors ${\vec{x}}_{global}$ and ${\vec{x}}_{relative}$ are defined as the refined initial coordinate and its corresponding relative coordinate, respectively. Because at least three images are needed for 3D reconstruction, and based on the consideration of computation complexity and efficiency, we set *K* to be 3 when implementing this module.

To sum up, we have introduced the detailed design of *SITE*, consisting of three main components, *Acoustic Localization*, *Decision Scheme*, and *Location Refinement*. In the following section, we present how to evaluate the performance of *SITE*, and make a comparative analysis of evaluation results.

## 5. Evaluation

### 5.1. Experiment Setting

To evaluate *SITE*’s performance, we built a prototype on the Android platform, which is compatible with any smartphone that includes a microphone, a Yei Technology motion sensor and a camera. For the *Acoustic Localization* module, we invoke android APIs to realize all the components, such as BPF and PLL. Meanwhile, we utilize the VSFM toolkit [18] to realize the function of achieving relative coordinates.

For SITE, each location is computed by the acoustic signals and the location of each anchor, thus no accumulative error exists in the localization result. Meanwhile, considering that a trajectory could be obtained by frequently localizing the user, we only conduct static location localization. Moreover, for a limited smartphone, a user’s trajectory can also be obtained by real-time tracking, combining with pedestrian dead reckoning and particle filtering; therefore, in this paper, we do not focus on a user’s trajectory.

**Figure 9:**
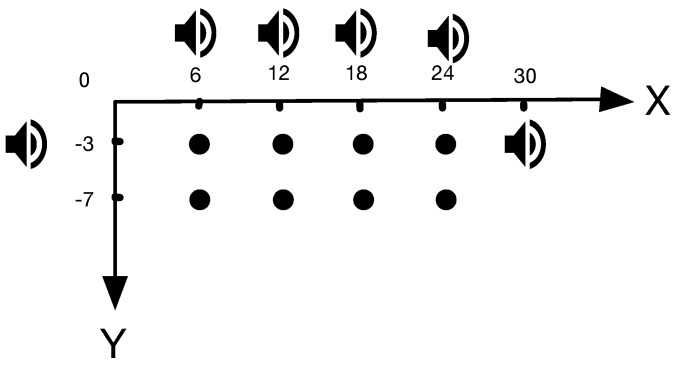
Acoustic Sources Settings in the evaluation.

In our evaluation, we used the same experimental deployment in different indoor environments, such as the lobby of a building (Section 5.2.1) and the library (Section 5.2.2). As Figure 9 illustrates, six phones were deployed in the floor plan as acoustic sources with a precise coordinate individually. In our setting, these coordinates were (0,−3), (6,0), (12,0), (18,0), (24,0), and (30,−3) (meters). Moreover, we set the central frequency of these acoustic sources to 17,000 Hz, 17,500 Hz, 18,000 Hz, 18,500 Hz, 19,000 Hz, and 19,500 Hz, respectively. In addition, we also chose eight spots at y∈{−4,−7} and x∈{6,12,18,24} as testing spots. As black point marked in Figure 7, we tested the prototype at eight locations, and the coordinates are (6,−3), (12,−3), (18,−3), (24,−3), (6,−7), (12,−7), (18,−7), and (24,−7), respectively. To acquire much more data for analysis, we repeated localization 40 times at each test spot. Finally, we comparatively analyzed *SITE* and other methods in localization accuracy.

### 5.2. *SITE*’s Performance

In an indoor environment, multi-path interference, materials used in walls, pedestrian walking around, the layout of anchors and even other factors will influence *SITE*’s performance. Therefore, to offer a comprehensive analysis of *SITE*’s performance, we evaluated the localization accuracy in different environments and compared it against other methods. Meanwhile, we also analyzes the impact of some key factors, such as the number of acoustic anchors and decision factor $\delta_{1}$. In the following, we give a detailed introduction.

#### 5.2.1. Lobby of a Building

First, aiming to compare against the proposed system in [23] and Swadloon [19], we conducted the evaluation at the deployment scenario illustrated above. As Figure 9 illustrates, the speaker and the solid black circle separately represent the acoustic source and the target point.

Method: For individual target, we first shake the phone at an arbitrary path for a while, and collect acoustic signals for computing location at the same time. According to the results of *Decision Scheme* mentioned above, if necessary, we took some photos using the phone’s camera for refining. We conducted the evaluation in line-of-sight (LOS) situation such as during off hours with little pedestrian foot traffic, and also in non-line-of-sight (NLOS) situation such as working time with pedestrian foot traffic. We compared our system against Swadloon and [23]. We collected results and performed a comparative analysis, including plotting CDF (Cumulative Distribution Function) of localization error.

**Figure 10:**
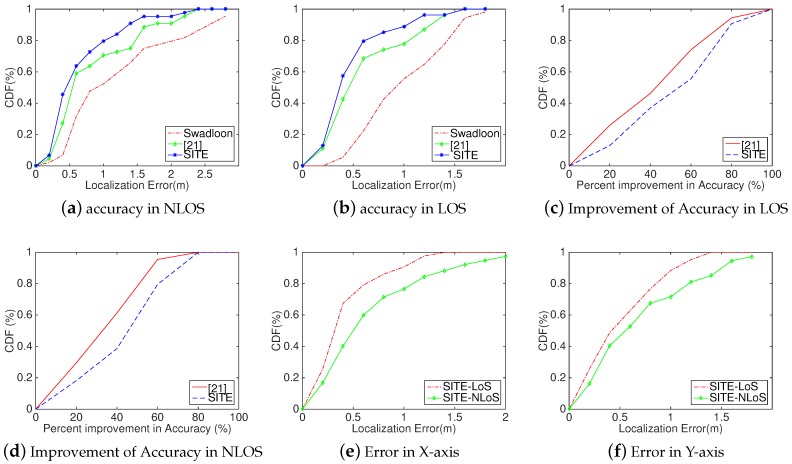
Evaluation Results of *SITE*, the method in [23] and Swadloon in the Lobby of a building: (**a**) CDFs of localization error in NLOS; (**b**) CDFs of localization error in LOS; (**c**) CDFs of improvement of localization accuracy in LOS; (**d**) CDFs of improvement of localization accuracy in NLOS; (**e**) CDF of localization error in X-axis; and (**f**) CDF of localization error in Y-axis.

Analysis: As shown in Figure 10, *SITE* is feasible and achieves a better performance than other methods on the same condition and experimental setup. In Figure 10a,b, we, respectively, compare *SITE*’s localization accuracy with two other methods (Swadloon and the method in [23]) in NLOS (Figure 10a) and LOS ((Figure 10b) condition. As expected, SITE achieves the best performance in these two conditions. In addition, we can also observe that *SITE* achieves a higher improvement in LOS than does in NLoS. This is due to changing of the indoor environment and the pedestrian movement in NLOS situation, the reconstructed 3D space using VSFM technique only with images is becomes less accurate than that in LOS situation, making the relative relationship inaccurate. Next, we analyzed the improvement of localization accuracy in different conditions and compared with the proposed method in [23]. In Figure 10c,d, we can intuitively see that SITE achieves a higher improvement in both conditions. The median improvement of SITE in LOS and NLOS condition respectively are 54.67% and 43.83%, which are 45.75% and 32.03% by [23]. According to our results in Figure 10e,f for localization error of X-axis and Y-axis, it is easily found that *SITE* has a better performance of localization accuracy in X-axis than its in Y-axis, such as *SITE* achieves a median error of 0.30 m and 0.40 m in X-axis in LoS and NLOS condition, which degrade to 0.42 m and 0.48 m in Y-axis, respectively. Here, this phenomenon of downgrading is probably because the layout has all anchors deployed on the same side in our experiments. Therefore, it is necessary to research on how the layout affects the performance of the localization method and the optimal approach to achieve the best performance.

In addition, we also comparatively analyzed SITE’s performance using numerical representation from three aspects, Median, Mean and Variance, as shown in Table 2. We can observe that SITE achieves a median localization error of approximately 39 cm and 42 cm in LOS and NLOS conditions, respectively, which decreases more than 3 cm over [23]. Besides, SITE also achieves a smaller mean localization error than [23]. To verify *SITE*’s robustness, we computed the variance of localization error in both conditions. As the fourth column of Table 2 lists, in LOS condition, SITE performs best and the variance is only 0.1 m.

#### 5.2.2. Library

Here, we test how *SITE* performs in a stressful environment full of many other acoustic signals and multi-path interferences.

Method: In this paper, we chose the library as our experimental scene. This is because there are many people walking around, many obstacles such as shelves with books that block the line from an acoustic source to the phone, and many multi-path interferences. The settings of acoustic sources are the same as above. At first, we collected the localization results at different testing spots as Section 5.1 describes. Then, we compared the results against Swadloon.

**Figure 11:**
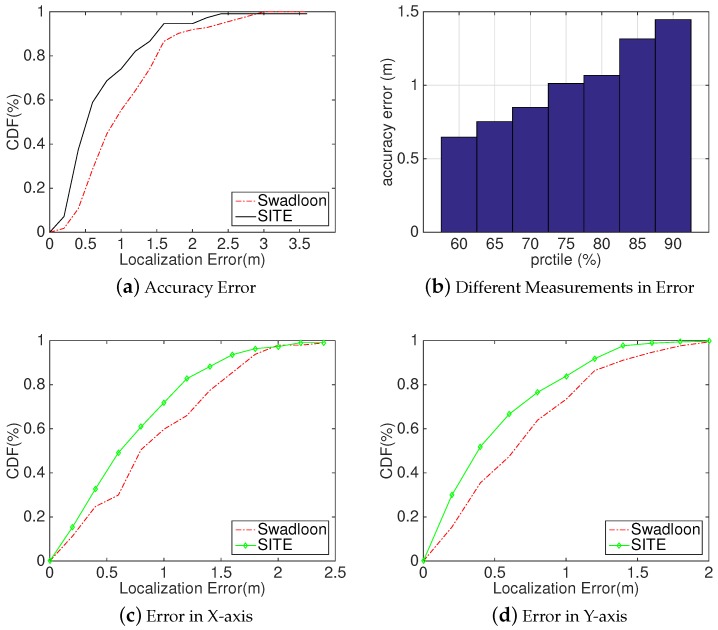
Evaluation Results in university’s library: (**a**) CDFs of localization error in library; (**b**) histogram of localization error in different percentile; (**c**) CDFs of localization error in X-axis; and (**d**) CDFs of localization error in Y-axis.

Analysis: As Figure 11 depicts, we separately computed the localization errors in distance (Figure 11a), X-axis (Figure 11c) and Y-axis (Figure 11d). From these figures, we can intuitively observe that *SITE* is still feasible in a stressful environment, and also performs better than Swadloon. For example, *SITE* achieves the localization error within 0.47 m, 0.85 m, and 1.44 m at the percentaged of 50%, 70%, and 90% respectively, whereas Swadloon degrades to 0.94 m, 1.32 m and 1.8 m, correspondingly. In contrast to Figure 10a,b, the line of improvement performs much more smooth than does in the lobby of a building. The reason for this phenomenon is that the environment of a library will not change frequently, results in much more concomitant features and acquiring much more accurate and stable reconstructed 3D space. Hence, *SITE* could have a stable performance on improving the localization accuracy. Furthermore, according to Figure 11c,d, *SITE* achieves a median error of 55 cm in X-axis and 48 cm in Y-axis compared to 76 cm and 68 cm in Swadloon.

#### 5.2.3. Impact of Number of Acoustic Sources

Method: According to the presented localization method in Section 4, at least five acoustic sources are needed to localize the user using our proposed *Decision Scheme* introduced in Section 4.3; by varying number of acoustic anchors, we could compare the localization performance of *SITE* in the following three situations: (a) localizing the user only relying on acoustic signals while there are three acoustic sources; (b) refining coordinate directly using VSFM as four acoustic sources are deployed; and (c) using our proposed localization scheme with at least five sources anchors. Therefore, we varied the acoustic sources from 3 to 6 for a comparative analysis of *SITE*’s localization performance.

**Figure 12:**
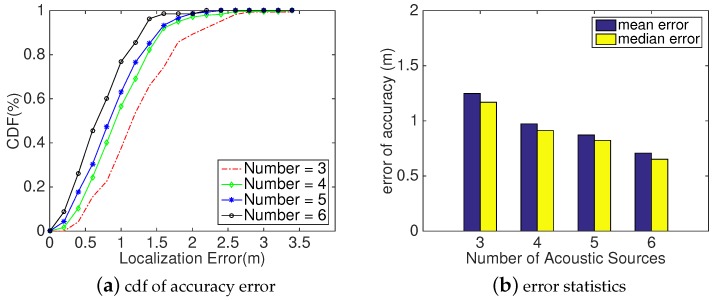
*SITE*’s Device Localization Accuracy measured against the number of acoustic sources. (**a**) plot cdfs of accuracy error at different numbers. (**b**) Two statistical measurements of performance: mean error and median error.

Analysis: In Figure 12, we can intuitively observe that the localization accuracy improves as the number of acoustic sources increases. When there are three acoustic sources deployed in the testbed and *SITE* localizes the user only relying on the acoustic signals as described in Section 4.2, we found that the median localization error is 1.17 m, which might result from the indoor interference factors having a great influence on the estimation. Adding one anchor in the testbed, we could see an obvious improvement in Figure 12a,b. However, it is out of our expectation that the localization performance changes very little when we used five acoustic anchor, according to our *Decision Scheme*, even though the decision module is activated when there are five anchors. Affected by indoor interference factors, *SITE* has a great chance to obtain an empty CL when the module *Location Refinement* is activated. In other words, most likely, *SITE* localizes the user by directly refining the coordinate estimated by acoustic signals with VSFM technique as *SITE* does with four acoustic sources. However, while we deploy six acoustic sources, *SITE* achieves a high improvement and the median localization error degrades to 0.63 m, as shown in Figure 12b. This might be because *SITE* has a higher likelihood to acquire the user’s localization directly from the *Decision Scheme* with non-null CL. Thus, by increasing the number of acoustic anchors, *SITE* could achieve a much better localization performance, but, correspondingly, it will result in much higher computation workloads. Because the energy consumption is a key factor for smartphone design, to balance the computation workloads and the localization accuracy, in our implementation, we only select six acoustic anchors to localize.

With the objective to observe the influence of number of acoustic sources on *SITE*’s localization performance, we evaluated how *SITE* performs by varying acoustic sources.

#### 5.2.4. Impact of Decision Factor $\delta_{1}$

Here, an evaluation on the accuracy performance of *SITE* under different values of *Decision Factor* $\delta_{1}$ is presented.

Method: As shown in Section 4.3.2, aiming to obtain more sets to find an optimal coordinate as one input of *Location Refinement* in Section 4.4, we brought in another threshold $\delta_{1}$ to mark all four-sized sets according to the decision scheme in Section 4.3. In our implementation, we set the threshold $\delta_{1}$ to 0.45 m as the mean localization error is likely less than 0.5 m. However, aiming to evaluate its impact on the accuracy of *SITE*, we chose $\delta_{1}$ as 0.4 m, 0.45 m, 0.5 m, and 0.6 m, respectively, and conducted the localization process to gather localization results. Then, we comprehensively analyzed the gathered locations.

**Figure 13:**
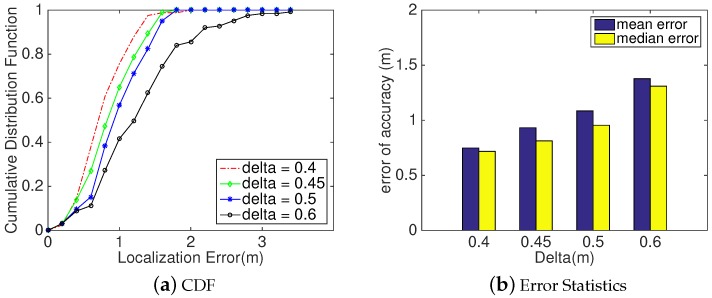
*SITE*’s Device Localization Accuracy measured against different values of *δ*_1_. (**a**) Plot CDF of localization error at different values. (**b**) Two statistical measurements of performance: mean error and median error.

Analysis: As illustrated in Figure 13a, we can intuitively observe that *SITE*’s performance decreases as *Decision Factor* $\delta_{1}$ increases from 0.4 to 0.6. This is because the larger $\delta_{1}$ is, the lower is the probability to activate *Location Refinement* module to correct the initial location. Moreover, we also confirm this observation from the aspect of median error depicted in Figure 13b. When $\delta_{1}$ is 0.4 m, 0.45 m, 0.5 m, and 0.6 m, the corresponding median errors are 0.72 m, 0.81 m, 0.95 m and 1.31 m, respectively. However, the difference of median errors between 0.5 and 0.6 is twice larger than that between 0.4 and 0.5. This phenomenon might be caused by the procedure of refining initial locations in module *Location Refinement*. In some situations, even though *SITE* has refined the initial locations to compute the user’s coordinate, its result is still not acceptable. To figure it out exactly, we further fourn the reason is *dirty data* among the initial locations. Here, *dirty data* represent locations that have much larger distance between majority of initial locations than others, such as the two upper-left red points in Figure 3b. Hence, erasing *dirty data* will have a positive influence on improving the localization accuracy of *SITE* and decrease its computational workloads.

#### 5.2.5. Overhead

The localization procedure of SITE can be separated into two phases: (i) computing initial location using acoustic signals and judging their stability and usability by module Decision Scheme; and (ii) refining the initial location only as the result of Decision Scheme is DIVERGED. As the latter phase depends on the performance of acoustic localization, and the direction finding is always running, we separately analyzed the SITE’s computation overhead in different phases. Here, we focus on the CPU usage of the phone.

In the first phase, while SITE processes a single acoustic signal to achieve its corresponding relative direction, we found the average CPU usage is 21.02%. Using more acoustic channels, the passband of BPF narrows, which results in higher computation overhead for the individual signal. In our evaluation, the average CPU usage increases up to 73.08% while *SITE* processes six signals at the same time. It will last approximately 3 s for the signal samples of 1 s on average. After achieving six relative directions, *SITE* computes coordinates using different subsets, as mentioned in Section 4.2.3, which continues for about 2 s. The average CPU usage performs a decrease by 37.6%. Then, *SITE* activates module *Decision Scheme*; during the procedure of searching for ConvergenceSet, the average CPU usage drops to 30.6% and it lasts less than 1 s. As we describe in Section 4.3.2, if we fail to obtain the user’s location, *SITE* will search an optimal coordinate as an input of the module *Location Refinement*, which takes approximately 1.5 s and the average CPU usage increases 2% from 30.6%. From the statistics, we can observe that the main cost for computation is in the phase of estimating relative directions. However, as we only shake the phone for a short duration, the overall computation is affordable as the computational ability of a smartphone rapidly increases.

For the refining phase, the majority of smartphones are equipped with GPU (Graphics Processing Unit), which greatly reduces that the computation overhead and time of processing pictures for relative locations. The CPU usage changes very slightly, by only 2% increase on average.

However, many techniques can efficiently reduce the energy consumption and time delay; for example, using a backend server to receive acoustic signals (or images if necessary) collected by smartphone and compute the user’s location. While dead reckoning technique is widely applied in indoor localization/tracking, it provides an alternative method to improve the localization efficiency at a cost of accuracy. As it is not in the scope of this work, we do not make a further analysis.

## 6. Discussion

In this section, we discuss some potential concerns with *SITE*, and point out some further work based on *SITE*.

*SITE* should be capable of removing the interference of invalid location. In Section 4.2, *SITE* calculates a set of temporary locations for deciding whether these locations converge closely to the true physical location. Nevertheless, some invalid locations where localization errors are extraordinarily large exist among these temporary results. However, these invalid results not only aggregate the computation burden but also result in location dilution of precision. Hence, removal of the invalid locations will make progress with the efficiency and accuracy of *SITE*.

*SITE* cannot estimate the object’s location in 3D space. As described in Section 3.1, *SITE* only considers 2D angle not 3D direction, and assumes the phone and acoustic anchors are approximately at the same height. Therefore, in the case of estimating an object’s 3D coordinate, this assumption is no longer suitable.

*SITE* can label the location with semantic information. Although *SITE* performs accurately and robustly, this location is directly represented by numerical values. However, the semantic information, linked to some specific places, functions, etc. that a user can understand intuitively, is much more valuable than the absolute coordinate values. Moreover, it can satisfy much more user demands. For refining location, *SITE* will require the user to take some photos to reconstruct a 3D space. Besides, we also can acquire environment information via image processing techniques, thus we can produce semantic information by associating with its coordinate. Meanwhile, we can generate a semantic map via crowdsourced automatic floor plan construction [36,37,38].

## 7. Conclusions

Aiming to accurately and stably achieve indoor location information, in this paper, we present a novel indoor localization scheme, *SITE*, that combines acoustic signals and images to localize the user. Through tracking the phone’s directions relative to the acoustic sources, *SITE* could obtain a set of locations corresponding to any *m* (m≥ 3) sources using the direction differences. Afterwards, it searches the largest ConvergenceSet for computing the user’s location. This is based on a key observation that has been proven in Section 3.2. While the simultaneously estimated locations using different sets of acoustic anchors are within a small circle, the results converge to a point near the true location. If no ConvergenceSet is returned, VSFM technique is utilized to extract the relative coordinates, each corresponding to a photo site, which also has a coordinate computed using acoustic signals. With these two pairs of coordinates, an optimal transformation relationship is achieved and used to estimate the user’s location. According to the evaluation results, we can find that SITE is excellent on the performance of localization accuracy, robustness, and feasibility in practical application.

## Figures and Tables

**Table 1:** Accuracy comparison with different localization systems.

System	Technique	Accuracy	Limitations
SpotFi [17]	WiFi, AoA, ToF	40 cm median	might not be scalable, high battery consumption, not suitable for real time localization
GuoGuo [2]	Acoustic Signals, TOF	6–25 cm median	requires customized beacons, cannot work in high sound pollution,
Swadloon [19]	Acoustic Signals	42 cm median	affected by sound pollution
ArrayTrack [3]	WiFi, AoA	23 cm median	requires some modifications to the AP, extra costs
[33]	Bluetooth, WiFi, Fingerprinting	2.33 m median	update periodically, time-consuming, labor-intensive
Zee [4]	WiFi fingerprinting, inertial sensors	3 m median	update periodically, time-consuming, labor-intensive

**Table 2:** Performance on median error, mean error and variance error.

Performance	Median (m)		Mean (m)		Variance	
	LOS	NLOS	LOS	NLOS	LOS	NLOS
Swadloon	0.9567	0.8844	0.9848	1.2005	0.1799	0.6598
[23]	0.4274	0.4523	0.5804	0.8150	0.1576	0.3761
SITE	0.3911	0.4228	0.4706	0.6519	0.099	0.2486

**Figure A1:**
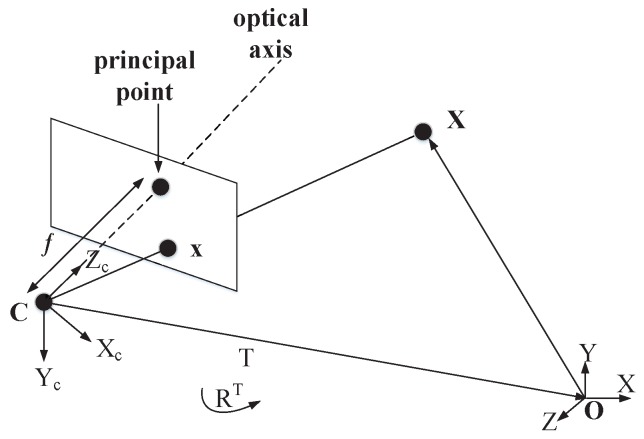
Pinhole projection of a 3D point X onto a camera image plane. The extrinsic parameters of the camera R,T represent the rigid body transformation between the world XYZ coordinate system and the camera $X_{c}Y_{c}Z_{c}$ coordinate system.

## Appendix A. Deduction of Equation (14)

Notation: For convenience, in this section, we use the style homogenous to represent a point. Therefore, an *N*-dimensional point will scale up to a (N+1)-element vector in homogenous. For example, a three-dimensional point $X={\left[\;X\;Y\;Z\;\right]}^{T}$ can be represented as its corresponding homogenous coordinate $\tilde{X}∼{\left[X\;Y\;Z\;1\right]}^{T}$.

To explain the principle of image projection, we choose a common camera model, *pinhole projection* depicted in Figure A1. According to *pinhole projection*, for a given three-dimensional point, we can project it to a 2D panel and obtain the corresponding coordinate. The whole procedure consists of three components: the rigi dbody transformation, the 3D to 2D transformation and the 2D to 2D transformation. We describe these three components in the following.

1. Rigid body transformation: As illustrated in Figure A1, for point X, we assume its coordinates in the world coordinate system(OXYZ) and the camera coordinate system($CX_{c}Y_{c}Z_{c}$) are ${\left[X\;Y\;Z\;1\right]}^{T}$ and ${\left[X_{c}\;Y_{c}\;Z_{c}\;1\right]}^{T}$, respectively. Then, a transformation (R, T) that relates $\tilde{X}$ to $\tilde{X_{c}}$ can expressed as:
  (A1) $$\left[\begin{array}{c} X_{c}\\ Y_{c}\\ Z_{c}\\ 1 \end{array}\right]∼\left[\begin{array}{cc} R & T\\ 0 & 1 \end{array}\right]\left[\begin{array}{c} X\\ Y\\ Z\\ 1 \end{array}\right]$$
  here, (**R**, T respectively are a 3×3 matrix and 3-element vector.
2. 3D to 2D transformation: As the coordinate $\tilde{X_{c}}$ is known, we project it to the camera image plane as the point x shown in Figure A1. Assuming $\tilde{x}∼{\left[x\;y\;1\right]}^{T}$, according to the triangle theory,
  (A2) $$x=f\frac{X_{c}}{Z_{c}}\;\;\;\;\;y=f\frac{Y_{c}}{Z_{c}}$$
  where *f* is the focallength. If we set *f* to be 1, then using homogenous coordinates $\tilde{X_{c}}$, we can get the 2D points $\tilde{x}$ by Equation (A3),
  (A3) $$\left[\begin{array}{c} x\\ y\\ 1 \end{array}\right]∼\left[\begin{array}{cccc} 1 & 0 & 0 & 0\\ 0 & 1 & 0 & 0\\ 0 & 0 & 1 & 0 \end{array}\right]\left[\begin{array}{c} X_{c}\\ Y_{c}\\ Z_{c}\\ 1 \end{array}\right]$$
  Because $\tilde{x}$ is defined only up to scale, it does not depend on the magnitude of $X_{c}$, i.e., it only relies on the relative direction between the 3D point and the camera.
3. 2D to 2D transformation: This transformation relates the point $\tilde{x}$ to a pixel coordinate $\tilde{u}∼{\left[u\;v\;1\right]}^{T}$. It can be denoted as :
  (A4) $$\tilde{u}∼K\tilde{x}$$
  where K, defined as Equation (A5), represents the camera calibration,
  (A5) $$K=\left[\begin{array}{ccc} \alpha_{u} & s & u_{0}\\ 0 & \alpha_{v} & v_{0}\\ 0 & 0 & 1 \end{array}\right]$$
  where $\alpha_{u}$, $\alpha_{v}$ and *s*, respectively, are two scale factors and a skew, and $u_{0}={\left[u_{0}\;v_{0}\right]}^{T}$ represents a principal point. As Figure A1 illustrates, it is an intersection of the optical axis and the camera’s image plane. Furthermore, both are the camera’s intrinsic parameters.

With the combination of Equations (A1), (A3) and (A4), the transformation relationship between the point $\tilde{X}$ and its pixel coordinate $\tilde{u}$ is as follows,

(A6) $$\tilde{u}∼P\tilde{X}$$

where $P∼K\left[\begin{array}{c} R\;T \end{array}\right]$ is a 3×4 projection matrix.

Provided that two projection matrices P and $P^{\prime }$ of two image pinhole are equal, a point $X^{\prime }$ in the coordinate system of camera $C^{\prime }$ is known, its corresponding location X in the coordinate system of C can be computed using Equation (A7):

(A7) $$X=RX^{\prime }+T$$
